# Incorporating engaged learning into medical student large group sessions

**DOI:** 10.15694/mep.2017.000120

**Published:** 2017-07-03

**Authors:** Kathryn S. Ivy, Allison R. Larson

**Affiliations:** 1 Boston University School of Medicine; 2 Boston University Medical Center / Boston University School of Medicine

**Keywords:** interactive learning, engaged learning, large group learning

## Abstract

This article was migrated. The article was marked as recommended.

**Purpose**: In recent years there has been increasing emphasis on active and engaged learning in medical education. The purpose of this study was to determine medical student satisfaction and performance in a module where 44% of the educational time was spent in a combination of large group engaged learning sessions and at-home modules.

**Methods**: Over two years a week-long dermatology course was transitioned to a format that included numerous large group interactive sessions. Course satisfaction results, exam performance, and a questionnaire on engaged learning were assessed.

**Results**: Overall course satisfaction improved from 94% to 99% of students ranking the course as good or excellent as did the percent of students who rated lecture delivery as engaging (88% to 98%). The percent of students responding that the module provided opportunities for collaboration among students rose from 50% to 92%. We took away a number of learning points from these sessions based on student feedback, including a need to be sensitive to the time required for pre-class learning modules and the format of such modules.

**Conclusions**: Based on student feedback, we found that large group teaching was effective in fostering collaboration as well as improving self-reported comprehension and overall course satisfaction.

## Introduction

“Active learning” is currently a standard for the Liaison Committee on Medical Education (LCME) ( “Functions and Structure of a Medical School,” 2015). Our institution has set a goal for each first and second year medical student course to gradually shift to 50% traditional lecture time. The remaining time would be spent on in-class active or engaged learning or on the associated at-home preparatory work for the engaged learning sessions. Interactive learning has been shown to yield positive results ( Gross et al., 2015; Neville, 2009), and therefore we decided to transition our dermatology course to a 50% engaged learning model, along the lines of our institution’s goal. Particularly in a clinical discipline, it can be challenging to coordinate leaders for numerous simultaneous small-group discussion sections as in a traditional flipped classroom approach ( Sharma et al., 2015). Therefore we decided to incorporate large group engaged learning sessions into our dermatology course. These sessions took place in an auditorium setting under the guidance of one or two faculty members. We sought to evaluate the success of this model measured by overall course satisfaction, exam performance, and a questionnaire on students’ experiences with active and engaged learning.

## Methods

Over the course of 3 years, a lecture-based dermatology course was transitioned to 56% traditional lecture time, which equated to 10 hours of lecture, 6.5 hours of engaged learning sessions, and 1.5 hours of at-home preparatory modules for in-class sessions. This course was given to the entire second year medical student class at our institution (approximately 180 students per year) as well as a group of first-year physician assistant students (approximately 25 students per year).

In the first year of the transition, 5 clicker-based audience response question sessions (3.9 hours total) were added for review of the lecture material. In the second year, 3 lectures were converted to condensed Powerpoint modules, 20-40 minutes in length, with voice-overs for at-home preparation, followed by in-class sessions on these topics. In the third year, these modules were reformatted as videos with interactive questions. One of the sessions was a clicker-based question and answer session while the other two involved breaking into small groups of 2-3 students within the auditorium to answer a set of problems of greater complexity than the material presented in the at-home modules (think-pair-share method). One or more faculty members circulated to answer questions while students worked on the problems. The answers were then reviewed with the entire group.

Outcomes were assessed through exam performance and a standard post-course survey including questions on overall satisfaction as well as satisfaction with the engaged learning portion of the course. Additionally, immediately following the dermatology module, the students were sent an anonymous questionnaire on their experience with interactive sessions during the first 6 months of the academic year.

## Results

Exam performance remained the same with a mean of 88%, standard deviation of 3.0 in the year before interactive sessions and in the fourth year a mean of 92% with standard deviations of 4.7 ( Table 1). Overall course satisfaction improved from 94% of students ranking the course as good or excellent to 99% all three years after the intervention. The percent of students who rated lecture delivery as engaging increased from 88% to 98% over four years. The percent of students responding that the module provided opportunities for collaboration among students rose from 50% to 92% across the four years with the introduction of the large group interactive sessions in the final year ( Table 1).

**Table 1.** Student satisfaction and exam scores over four years

	2013	2014	2015	2016
Average exam score ± standard deviation	88 ± 3.0	90 ± 6.3	90 ± 5.5	92 ± 4.7
% students rating course as good or excellent	94	99	99	99
% students agree that lecture delivery is engaging	88	94	96	98
% students agree that module provided opportunities for collaboration between students	50	59	91	92

An anonymous questionnaire was sent out to the second-year medical students at the end of the week-long dermatology module after the second year of the intervention. Questions were framed to promote honest feedback supported by students’ personal experiences. All courses in the first half of the second year medical student curriculum had begun to incorporate a small number of large group interactive sessions in various formats. In analyzing the survey responses we developed categories of concerns and praises from students regarding these experiences with active learning. The term ‘flipped classroom’ was used in the survey to describe a large group auditorium-based session focused on discussion and application of concepts learned independently prior to class. We evaluated student responses to the following question: “Why are flipped classrooms effective or ineffective?” Results were categorized into three primary themes: time, content and session structure ( Table 2). Student responses of pros and cons are listed in Table 3.

**Table 2.** Student feedback on interactive sessions

Major categories	Associated terms and concepts
Time	Amount of pre-work; efficient use of time; attendance
Content	Visual topics; quantitative topics; subjects with less dense information; didactic vs. flipped classroom; memory aiding; condensed pre-work; concept application
Session structure	Repetition; case-based; clicker question/answer-based; conversationally-based; leading questions to stimulate discussion; location; faculty discussant; peer teaching; optimal number of students for proper collaboration

Out of all categories, the most commonly referenced was ‘time’ ( Table 3). This is not surprising as efficient learning is a priority of medical students as they balance clinical experiences, didactic curricula and preparation for board examinations. Many students praised condensed pre-class videos of 15-30 minutes in length as key to learning efficiently and effectively in the dermatology module. More extended pre-class video material, in the range of 45-120 minutes, was felt to be too long. One student commented, “Having to watch several extra hours of online video became quickly burdensome in an already busy schedule”. Another student observed, “It’s difficult to prep in time, and if you don’t have time to prepare, you don’t get as much out of the [flipped] class”. An additional learning point in structuring pre-class material with efficiency in mind was that our students strongly preferred a video format to voice-over Powerpoint so they could have multiple windows open on their computer simultaneously to facilitate note-taking.

**Table 3.** Pros and Cons of each category

Category	Pro	Con
Time	- No required attendance - Can watch video of interactive session at 1.5-2X speed to save time	- Too much pre-class material - Flipped session after exam - Not enough time to prepare adequately - Less efficient than self-study
Content	- Process information better by thinking out loud- Visual topics lend themselves to image-based questions - Quantitative topics lend themselves to case -based problems - Sessions cover many entities instead of one small concept/disease	- For complex concepts, not enough time before session to absorb material and be prepared to apply it - No conceptual difference between pre-class work and in-class experience
Session structure	- Clickers offer independent learning and group collaboration - Every student goes through every problem/case - Faculty explanations - Explicit session goals promote directed learning - Case-based aids in retention - Pre-class material in video format	- Auditorium a less effective setting for collaboration - Splitting up cases where students review only some- Student explanations can be circuitous - Not enough student attendance for proper collaboration

Student responses regarding content of the active sessions suggested visually-oriented disciplines (i.e. dermatology) and quantitative topics (i.e. biostatistics) were particularly helpful to review in a large group interactive format. Within the dermatology module, image-based questions allowed students to test their ability to recognize skin diseases and the associated histologic patterns out of the context of a lecture slide and in a variety of presentations. These sessions also helped students begin to integrate information between lecture topics. For quantitative topics, such as public health, flipped classrooms were structured with a pre-class video regarding a statistical concept (i.e. sensitivity and specificity) and class time was used to practice applying these concepts and determine what the results meant in terms of counseling a patient. In both of these areas, students found a large group interactive learning method particularly useful in pointing out their misunderstandings of the material. Many felt that working on questions in groups of 2-4 students during these sessions (as opposed to 7 or more) gave all students within a group ample chance to speak and maximized learning.

While we did not detect a significant difference in exam performance, the free-response survey indicated that engaged learning affected students’ perception of their own comprehension. Students remarked their self-measured comprehension of the material improved after discussing with 1-3 other students. They felt the audience-response clicker questions were an effective way to test their knowledge because “class time was used to practice distinguishing between many terms and diagnoses” and they also “served very well to test our memory and highlight gaps in our knowledge or any questions we had.” These sessions provided an environment for students to work through confusing topics and to have conversations about the material with their peers and faculty members.

## Discussion

The flipped classroom is a current popular method of incorporating active learning for high school, undergraduate, and graduate education. Prober and Heath (2012) describe the ability of flipped classrooms to seize curiosity through student engagement of the material. This method gives the student responsibility for their learning before class, encouraging accountability and offering opportunities to dive deeper into the information past the standard curriculum ( Belfi et al., 2015; Prober & Khan, 2013). In addition, all active learning exercises work to engage the students with each other, offering opportunities for collaboration between peers ( Morgan et al., 2015).

Our comment-based results are derived from 6 months of experience incorporating interactive learning into the second year medical school curriculum with a specific focus here on the dermatology module. In a traditional flipped classroom as described by Salaman Khan, the ideal ratio of students to teachers varies from 4:1 to 25:1 ( Prober & Khan, 2013). This is different than our intervention where 1-2 faculty members worked with 50 to 60 students. This difference in student-to-faculty ratio necessitated a format where there was less individual interaction between teachers and students than would be possible in a smaller group. Thus many of the sessions in this module were audience response question-based or involved students working in small groups on questions followed by faculty-guided review of the answers with the entire class simultaneously.

Previous studies have assessed various aspects of the flipped classroom model including but not limited to student satisfaction, student improvement, and class attendance. Within the dermatology module, we saw an increase in attendance as the course progressed (measured through observation) and also an increase in student satisfaction, however there was no significant increase in exam scores, an observation seen in similar studies ( Liebert et al., 2016; Morgan et al., 2015). In addition to student satisfaction, faculty satisfaction has been suggested to increase with this learning format where faculty are freed from reiterating material and allowed to facilitate higher-order thinking and application of this material ( Mehta et al., 2013).

Our experience in the large group setting parallels previously published research on traditional flipped classrooms and the factors that most influence their success are time and content. Veeramani et al. commented on the time and resources it takes for instructors to prepare for these sessions, as well as the need for technological investment ( Veeramani et al., 2015). While advanced technology can certainly enhance interactive learning, we have found that successful sessions can be created using simple existing platforms. Time is important from the perspective of the learner as well and has been replicated as the major concern in many other flipped classroom studies ( Khanova et al., 2015; Prober & Heath, 2012; Prober & Khan, 2013; Whelan et al., 2016). Our survey results spoke to the importance of condensed pre-class work. Interactive learning entails a larger up-front time commitment on the learner’s part with the need to complete pre-class work, attend class, and then review the material afterward for further concept solidification ( Sharma et al., 2015). Lage et al. astutely observed that despite the extra work upfront, these sessions may be saving time if instructors are able to pinpoint the inconsistencies and mistakes in a student’s thinking and understanding of a concept ( Lage et al., 2000). A goal of the interactive dermatology sessions was to help students think critically and differentiate between similar-appearing conditions, sometimes discussed in different lectures. Additional unanticipated areas of confusion were brought to light by the audience response system as well as individual questions. These were discussed as they arose. These opportunities not only allowed the instructor to clarify confusing information but informed future improvements to the pre-class material ( Moffett, 2015).

Attendance has been a recent area of change and subsequent point of contention in medical education. More schools are offering recorded lectures, granting students flexibility in their study schedules with a resultant decrease in attendance. Flipped classroom sessions offer an enhanced learning opportunity through student engagement with each other, the material and their instructors. We observed a steady increase in attendance every day in this module, as did instructors at other institutions as they increased the number of flipped classroom sessions ( Prober & Heath, 2012). While attending class is not the most effective learning approach for every student, interacting with other students is something that cannot be done through one’s computer screen. Particularly in medicine, where interacting with our colleagues is critical to providing the best patient care, communication and the ability to work with others are key skills for medical students to gain prior to the clinical years.

We have focused on a visually-based discipline here, however, other groups have found this pedagogy to be effective in topics ranging from neuroanatomy to renal pharmacotherapy to biochemistry to the brachial plexus ( Nematollahi et al., 2015; Pierce & Fox, 2012; Prober & Heath, 2012; Veeramani et al., 2015). In these papers, the presentation style of the pre-class work appeared to be the most important variable that influenced student satisfaction. One key point of feedback that was reiterated by numerous survey responses from our students was the importance of a video format for pre-class work that would allow students to watch the video while concurrently taking notes on their computers. Another point of feedback we noted in our survey results was that students had greater confidence in their understanding of the material. This is not surprising as Jensen (2015) points out the improvements in student comprehension “may simply be the fruits of active learning” (pg. 1) not the flipped classroom format per se. Our experience echoes this finding in that, even in a large group setting, interactive learning improved subjective reports of comprehension.

In conclusion, we found that large group interactive learning sessions were effective in engaging students, aiding their comprehension of the material, and improving overall course satisfaction. The time required and format of pre-class work were of particular importance to students, similar to what has been found in traditional flipped classrooms. This format could allow for greater incorporation of interactive learning into classrooms without the need for numerous simultaneous small-group discussion leaders, thus allowing for a larger percentage of curricular time spent learning interactively.

## Take Home Messages

• Large group interactive sessions can successfully engage students in material
• Course satisfaction improved with replacement of traditional lecture with large group interactive sessions
• Time committment and format for pre-class work strongly influenced students’ perceptions of these interventions
• Large group sessions could allow for increased time spent in engaged learning regardless of the number of teaching faculty available

## Notes On Contributors

Dr. Allison R. Larson is an Assistant Professor of Dermatology and an Assistant Dean for Academic Affairs at Boston Medical Center / Boston University School of Medicine.

Kathryn S. Ivy is a third-year medical student at Boston University School of Medicine.
