# Supplementary material for: Incorporating engaged learning into medical student large group sessions
Source: MedEdPublish (2016). 2017 Jul 3;6:120. [Version 1] doi: 10.15694/mep.2017.000120 (PMC10885274; doi:10.15694/mep.2017.000120)
Supplement: Peer review history

# Reviewer report: Reviewer response for version 1

**DOI:** 10.21956/mep.19306.r28155

**Published:** 2017-07-12

**Authors:** P Ravi Shankar

**Affiliations:** 1 American International Medical University

This review has been migrated. The reviewer awarded 4 stars out of 5 As a strong proponent of active, small group learning strategies I enjoyed reading this paper. As mentioned in the paper by the authors and in the comments provided by the other reviewers, the evidence for the effectiveness of active, engaged learning strategies continues to grow. In most developed nations, faculty, students and the school administration are favorably disposed to small group learning. However, in many schools in developing countries and in many Caribbean medical schools, faculty and in many cases, the academic leadership are ambivalent toward these methods and continue to believe, despite evidence to the contrary, that students cannot learn on their own unless they are ‘taught’ by the teacher. I have been working to change this approach for most of my academic career. With increasing availability of technology and decreasing cost, faculty in developing nations can more easily post learning material online. Power point voice overs are not resource intensive, though, recording software to create video lectures may be expensive for some medical schools and settings. The authors’ use of team-based learning reduces faculty requirements and improves cost-effectiveness. Online learning platforms are becoming increasingly common in many areas of the world and have an important role to play in encouraging active learning. Looking back I wish these technologies and teaching-learning approaches were present when I went to medical school in the late 1980s-early 1990s. Many recent studies have also explored the impact of recorded lectures, video lectures and flipped classrooms on student attendance and the present study demonstrates how engaged learning improved student attendance and motivation. Interesting points have also been raised by other reviewers.

Reviewer Expertise:

NA

No decision status is available

# Reviewer report: Reviewer response for version 1

**DOI:** 10.21956/mep.19306.r28157

**Published:** 2017-07-07

**Authors:** James Fraser

**Affiliations:** 1 Griffith University

This review has been migrated. The reviewer awarded 3 stars out of 5 The author’s work in this paper supports previous publications regarding the educational benefits of interactive learning sessions. The ability for teaching staff to develop the resources necessary for these sessions with limited additional investment in technology is an important point for programs considering adopting a flipped model. From a teaching and learning view the ability to not only clarify misunderstandings of material but to be able to readily modify the learning materials from this feedback is important, and this can be difficult to achieve using a large group lecture delivery mode. Considering attendance as a maker for student engagement can sometimes be misleading but gaining an understanding from the students as to why this occurred could add to this observation. The author’s point about collegial interaction as one part of the hidden curriculum for group leaning is important. It would be interesting to see if there is an end point to continuing to remove lectures and replacing the format totally with a flipped interactive model.

Reviewer Expertise:

NA

No decision status is available

# Reviewer report: Reviewer response for version 1

**DOI:** 10.21956/mep.19306.r28156

**Published:** 2017-07-06

**Authors:** Trevor Gibbs

**Affiliations:** 1 AMEE

This review has been migrated. The reviewer awarded 3 stars out of 5 An interesting paper that although not adding a tremendous amount of new knowledge to our medical education toolbox, does raise some interesting points about making learning more active and participatory.As I was reading the paper I wondered why the authors stopped their innovations at that particular point. Should we start pushing the boundaries even further and try and eliminate lectures completely, make subjects which are a mixture of usual differentiation and knowledge more a combination of directed self learning, self study and explanatory sessions- just a thought.

Reviewer Expertise:

NA

No decision status is available
